# Interpretable Machine Learning for Predicting Radiation-Induced Hypothyroidism in Head and Neck Cancer: A Dual-Center Retrospective Study

**DOI:** 10.3390/cancers18172838

**Published:** 2026-09-02

**Authors:** Wenhui Li, Ying Zhang, Yangyang Ji, Rui Jian, Zequn Jia, Ziyu Wei, Yingjun Liu, Hua Zhou, Hongyang Yu

**Affiliations:** 1 Department of Radiation Oncology, The Second Affiliated Hospital of Harbin Medical University, Harbin 150086, China; liwh@hrbmu.edu.cn (W.L.); 202501404@hrbmu.edu.cn (Y.Z.); jianrui@hrbmu.edu.cn (R.J.); axbb@hrbmu.edu.cn (Z.J.); weiziyu@hrbmu.edu.cn (Z.W.); southerncross@hrbmu.edu.cn (Y.L.); 2 School of Computer and Big Data, Heilongjiang University, Harbin 150080, China; 3 Department of Radiation Oncology, The First Affiliated Hospital of Kunming Medical University, Kunming 650032, China

**Keywords:** head and neck cancer, radiation-induced hypothyroidism, machine learning, interpretability, SHAP, LightGBM

## Abstract

**Background/Objectives**: Radiation-induced hypothyroidism (RIHT) is a frequent complication in head and neck cancer (HNC) patients following radiotherapy, significantly impacting quality of life. This study aimed to develop and validate an interpretable machine learning model for predicting RIHT, with a focus on providing individualized risk assessment. **Methods**: The study included a development cohort (*n* = 256) from the Second Affiliated Hospital of Harbin Medical University and an independent external validation cohort (*n* = 296) from the First Affiliated Hospital of Kunming Medical University. Using 18 predictive features, six machine learning algorithms—Random Forest, Support Vector Machine, Multi-Layer Perceptron, Logistic Regression, XGBoost, and LightGBM—were constructed. Model performance was evaluated using AUC, balanced accuracy, F1 score, PR-AUC, Brier score, and Brier skill score. SHapley Additive exPlanations (SHAP) was applied for model interpretability. **Results**: In internal validation, LightGBM achieved the highest performance (AUC = 0.872, balanced accuracy = 0.703, PR-AUC = 0.712, and Brier skill score = 0.371), outperforming logistic regression. In external validation, Random Forest achieved the highest AUC (0.769), while XGBoost and LightGBM achieved AUCs of 0.721 and 0.716, respectively. SHAP analysis identified pre-treatment thyroid volume, T-stage, tumor site, mean thyroid dose (Dmean), and V50 as the most influential features for model prediction. Notably, dose–volume parameters (V30–V60) were highly correlated (r = 0.75–0.98); therefore, they should be interpreted as a cluster rather than as independent predictors. We caution against overinterpreting individual Vx thresholds as rigid clinical cutoffs. **Conclusions**: Machine learning models, particularly tree-based ensemble methods (LightGBM and XGBoost), demonstrate good predictive performance for RIHT. Combined with SHAP analysis, these models provide transparent, individualized risk assessments. However, the dose–response relationship for RIHT is continuous, and no single dose threshold should be used as a rigid clinical cutoff. Model code is available from the corresponding author upon reasonable request. Future prospective studies with time-to-event analysis are needed to further validate clinical utility.

## Simple Summary

Radiotherapy is a vital treatment for head and neck cancer, but it frequently causes permanent damage to the thyroid, leading to a condition called hypothyroidism that severely reduces patients’ quality of life. This study aims to create an intelligent computer program using machine learning to accurately predict which patients are at high risk of developing this condition using clinical characteristics and planned radiotherapy dose information. By analyzing clinical and radiation dose data from two medical centers, we identified key factors contributing to model predictions, such as the initial thyroid volume and radiation dose exposure. This research provides doctors with a personalized tool to achieve early identification of high-risk patients and adjust treatment plans to protect their thyroid function. For the research community, it demonstrates how artificial intelligence can make cancer care safer and more tailored to individual patient needs.

## 1. Introduction

Head and neck cancer (HNC) is the seventh most common cancer worldwide, with over 660,000 new cases and approximately 325,000 deaths reported annually [1,2]. Among these, head and neck squamous cell carcinoma accounts for about 4% of all malignant tumors, and its incidence remains high [3]. Radiation therapy is a cornerstone of comprehensive treatment for HNC. For patients with early-stage (Stage I or II) HNC, treatment typically involves surgery or radiation therapy. For patients with locally advanced disease, radiation therapy or concurrent chemotherapy can effectively destroy tumor cells and improve local control rates and overall survival [4].

Radiation-induced hypothyroidism (RIHT) refers to a condition in which, following neck radiation therapy for HNC, radiation damage to the thyroid gland impairs thyroid cell function, potentially leading to decreased or increased secretion of thyroid hormones. This can result in hypothyroidism (HT), hyperthyroidism, or even thyroid tumors [5,6]. Among these, RIHT is the most common. Studies indicate that among HNC patients undergoing radiotherapy, the incidence of RIHT can reach as high as 20–50%, and the risk persists for several years following treatment [7,8,9]. HT can lead to a decrease in metabolic rate and sympathetic nervous system activity, and may even result in a thyroid crisis, which seriously compromises the patient’s health. Once HT develops, patients may experience a range of clinical symptoms, including fatigue, sensitivity to cold, weight gain, low mood, and cognitive decline. These symptoms not only significantly impact the patient’s quality of life and well-being after recovery but may also increase the risk of cardiovascular disease due to a slowed metabolism, and could potentially affect subsequent cancer treatment and follow-up care [10,11,12].

Currently, most clinicians rely on periodic blood tests to monitor thyroid function following radiation therapy to predict and diagnose RIHT. However, this passive monitoring approach has a significant time lag; by the time laboratory values show abnormalities, patients may already be experiencing clinical symptoms. In recent years, machine learning (ML) has been applied to develop medical predictive modeling tools. Compared to traditional statistical models based on univariate analysis or logistic regression, machine learning models can account for complex interactions among clinical variables and capture intricate nonlinear relationships between them, thereby offering the potential to build models with superior predictive performance [13,14]. However, many high-performance machine learning models are often viewed as “black boxes” with decision-making processes that are difficult to understand, which severely hinders their acceptance and application in clinical practice. Recent advances in interpretable artificial intelligence methods can now be used to explain these models, and these interpretable machine learning models hold great potential for promoting the clinical application of machine learning in patient care [15].

This study aims to develop and validate an interpretable machine learning model for predicting RIHT in patients with HNC. We plan to integrate patients’ clinical characteristics, tumor site, detailed radiotherapy dose–volume parameters, and laboratory test data to construct a highly accurate predictive tool. We will use SHAP (SHapley Additive exPlanations) interpretable machine learning technology to interpret the model’s predictions and quantify the contribution of each feature to individual prediction outcomes. We expect this model to identify important predictive factors associated with RIHT following radiotherapy for HNC, facilitate the identification of high-risk patients, provide clinical decision support, and ultimately contribute to improving the long-term quality of life of patients with HNC.

## 2. Materials and Methods

### 2.1. Study Population

This study is a retrospective observational study conducted at two tertiary hospitals in China: the Second Affiliated Hospital of Harbin Medical University and the First Affiliated Hospital of Kunming Medical University. Data collected from September 2022 to December 2025 at the Second Affiliated Hospital of Harbin Medical University were used for model training and internal validation. Data collected from September 2020 to December 2023 at the First Affiliated Hospital of Kunming Medical University were used for external validation.

### 2.2. Inclusion and Exclusion Criteria

Inclusion criteria: Adults aged 18 years and older; patients with a pathological diagnosis of nasopharyngeal cancer, nasal cavity cancer, laryngeal cancer, oropharyngeal cancer, oral cavity cancer, or salivary gland cancer; treatment-naïve patients; patients who received intensity-modulated radiation therapy (IMRT); patients with complete clinical records, including demographic information, clinical variables, and radiation dosimetry; and patients with pre-treatment thyroid volume measurements and one year of follow-up on thyroid function.

Exclusion criteria: Patients with hypothalamic–pituitary abnormalities, thyroid dysfunction, severe hypertension, heart disease, or liver or kidney damage; cases with completely missing data on age, gender, or thyroid function. Patients who were lost to follow-up or who died before completing the 12-month thyroid function assessment were excluded. Thus, all included patients had complete 12-month follow-up data with no missing outcome information.

### 2.3. Data Collection and Preprocessing

This study constructed models using radiotherapy dosimetry and clinical variables, including only features tested at all study centers to ensure data consistency and comparability.

A total of 18 variables were ultimately included: demographic variables (gender and age); pre-treatment thyroid volume; thyroid dosimetric parameters, including Dmean, V20, V25, V30, V35, V40, V45, V50, V55, and V60; and clinical variables, including clinical stage, T stage, N stage, chemotherapy status, and tumor site. Vx represents the percentage of thyroid volume receiving a dose of at least x Gy. These dosimetric parameters were analyzed as continuous variables, and no specific threshold was predefined as a rigid biological cutoff. No imputation was performed because all included patients had complete data for the 18 selected variables.

### 2.4. RIHT Definition

This study focuses on patients’ thyroid function. Patient data were grouped based on the presence or absence of hypothyroidism following radiotherapy, and thyroid function data were collected before radiotherapy and at 3 and 12 months post-treatment. Serum samples were collected to measure the levels of free triiodothyronine (FT3), free thyroxine (FT4), and thyroid-stimulating hormone (TSH) in the serum. Reference ranges for the observed indicators were 0.81–1.89 ng/dL for FT4 and 0.380–4.340 mIU/L for TSH. Clinical hypothyroidism is defined as TSH levels above the normal range (>4.340 mIU/L) and FT4 concentrations below the normal range (<0.81 ng/dL); subclinical hypothyroidism is defined as TSH levels above the normal range (>4.340 mIU/L) and FT4 concentrations within the normal range.

This study used a binary outcome (RIHT vs. euthyroid) assessed at 12 months post-radiotherapy. We did not perform a time-to-event analysis because: (a) thyroid function was measured at protocol-specified intervals (baseline, 3 months, and 12 months) rather than continuously, precluding precise determination of onset time; (b) patients with disease progression or death within 12 months were excluded to ensure that hypothyroidism could be attributed primarily to radiation exposure rather than other clinical confounders. Consequently, competing risks such as death prior to RIHT were not applicable to the analyzed cohort.

To clarify the classification rules:

(1) RIHT endpoint: The primary endpoint was thyroid function status at 12 months post-radiotherapy. Patients with TSH > 4.340 mIU/L at 12 months were classified as RIHT, regardless of whether they met criteria for clinical or subclinical hypothyroidism, as both conditions reflect radiation-induced thyroid injury.
(2) Transient TSH elevation: Patients with TSH > 4.340 mIU/L at 3 months but normal TSH at 12 months were classified as euthyroid, because our primary endpoint was 12-month status and transient dysfunction does not represent persistent RIHT.
(3) Thyroid hormone replacement: No patient in our cohort initiated thyroid hormone replacement therapy before the 12-month assessment, as this was an exclusion criterion to ensure unbiased outcome classification.
(4) Central hypothyroidism: We acknowledge that patients with nasopharyngeal carcinoma receiving radiotherapy to the hypothalamic–pituitary region may develop central hypothyroidism (low FT4 with inappropriately normal/low TSH). Our definition based on TSH elevation would not capture this entity. We have added this as a limitation in Section 4.

### 2.5. Model Development and Model Interpretability

During model development, data from the Second Affiliated Hospital of Harbin Medical University (*n* = 256) were used as the development cohort. Internal validation was performed using 5-fold cross-validation repeated three times. In each repetition, the development cohort was partitioned into five folds, with four folds used for model training and the remaining fold used for validation; this procedure was repeated until each fold had served as the validation fold. Model performance was summarized across the cross-validation folds. Data from the First Affiliated Hospital of Kunming Medical University (*n* = 296) were retained as an independent external validation cohort and were not involved in model development.

Predictive models were constructed using the 18 predefined variables and six machine learning algorithms: Random Forest (RF), Support Vector Machine (SVM), Multi-Layer Perceptron (MLP), Logistic Regression (LR), XGBoost, and LightGBM. Model performance was evaluated using multiple complementary metrics, including the area under the receiver operating characteristic curve (AUC), accuracy, precision, recall, F1 score, balanced accuracy, area under the precision–recall curve (PR-AUC), Brier score, and Brier skill score [16]. The DeLong method was used to statistically compare the AUCs among different models [17].

All statistical analyses and machine learning model implementations were performed using R version 4.3.2. Data manipulation was conducted using the dplyr and tidyr packages. Machine learning models were implemented using the caret framework together with the corresponding model-specific packages, including xgboost and lightgbm. Model interpretability analyses based on SHapley Additive exPlanations (SHAP) were performed using the fastshap and shapviz packages. Data visualization was performed using ggplot2. Continuous variables are presented as mean ± standard deviation (SD), and their distributions were assessed using the Kolmogorov–Smirnov (K–S) test. For normally distributed continuous variables, comparisons between two groups were performed using the independent-samples *t*-test, whereas non-normally distributed continuous variables were compared using the Mann–Whitney U test. Categorical variables are presented as numbers and percentages and were compared using the chi-square test or Fisher’s exact test, as appropriate. A two-tailed *p*-value < 0.05 was considered statistically significant.

### 2.6. Hyperparameter Configuration

All machine learning models were initially configured using the default hyperparameters of their corresponding R implementations. The default parameter settings for each model are provided in Supplementary Table S2.

To assess the stability of these default parameters, 5-fold cross-validation repeated three times was performed within the Harbin development cohort. The cross-validation AUCs showed low variability (standard deviation < 0.03 for all models), indicating relatively stable performance across folds and repetitions. Given this stability and the modest size of the development cohort (*n* = 256), we opted not to perform extensive grid search to reduce the risk of overfitting.

For completeness, we conducted a limited grid search on the best-performing LightGBM model, exploring the number of boosting iterations (50, 100, and 200) and maximum tree depth (3, 5, and 7). This resulted in only a marginal AUC improvement (+0.01) compared to the default configuration. Therefore, default parameters were retained for all models to ensure reproducibility and simplicity. For SVM and MLP, which are sensitive to feature scaling, continuous predictors were standardized to have a mean of 0 and a standard deviation of 1 prior to model training.

## 3. Results

### 3.1. Patient Characteristics

**Figure 1:**
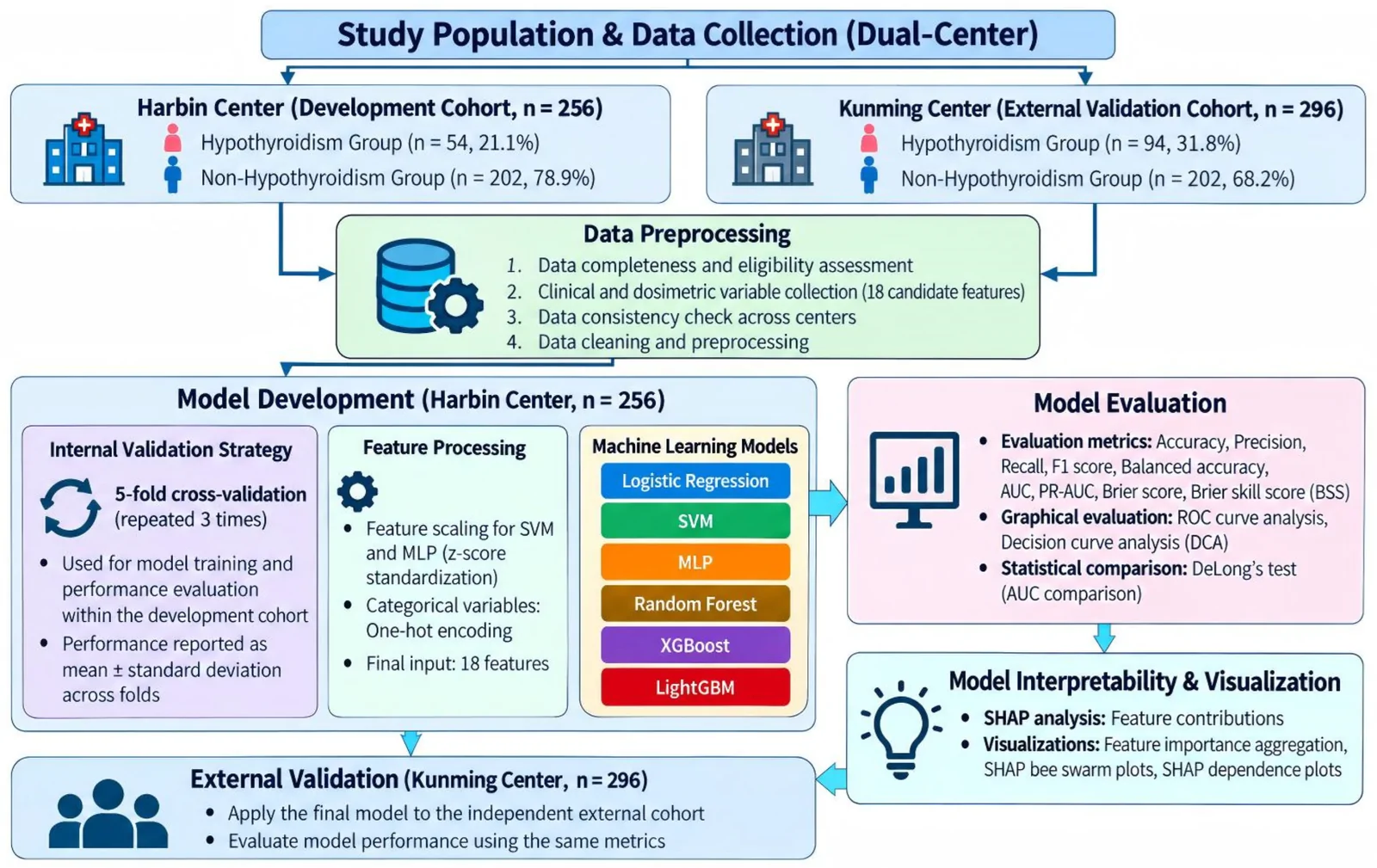
Research flowchart. Data from patients from two hospitals for model training, internal validation, and external validation.

The overall study design process is illustrated in Figure 1.

In this study, we conducted a descriptive analysis of the demographic and clinical characteristics of 256 patients from the Second Affiliated Hospital of Harbin Medical University. The research subjects included 74 patients with nasopharyngeal carcinoma, 110 patients with laryngeal cancer, 28 patients with oropharyngeal cancer, 29 patients with nasal cancer, 9 patients with salivary gland cancer, and 6 patients with oral cancer. Among them, there were 195 males and 61 females. There were 54 patients in the hypothyroidism group, with an average age of 58.04 ± 9.34 years. There were 202 patients in the group with normal thyroid function, with an average age of 58.75 ± 11.43 years. 21.09% (54/256) of all patients developed radiation-induced hypothyroidism (RIHT). The average thyroid volume of 54 patients with RIHT before radiotherapy was 11.72 ± 2.70 cm^3^; 78.91% of patients did not experience hypothyroidism (202/256), and the average thyroid volume before radiotherapy in the group with normal thyroid function was 13.21 ± 2.69 cm^3^. Our results indicate a statistically significant association (*p* < 0.001) between different thyroid volume groups and the incidence of RIHT.

Thyroid volume, thyroid Dmean, clinical stage, and tumor site were significantly associated with RIHT (Table 1), whereas T stage and N stage did not reach statistical significance in the univariate comparisons.

#### Correlation Among Dose–Volume Parameters

As expected, the dose–volume parameters (V20–V60 and Dmean) showed high inter-correlation. Supplementary Figure S1 presents the Spearman correlation matrix, with correlation coefficients ranging from 0.75 to 0.98 among V30–V60. Due to this high multicollinearity, the individual SHAP values for specific Vx thresholds (e.g., V50 vs. V55) should be interpreted cautiously. Therefore, we focus on the cluster of mid-to-high dose–volume parameters as a group rather than singling out individual thresholds. Thyroid Dmean, while also correlated with Vx parameters, provides a complementary summary of overall thyroid dose exposure.

### 3.2. Model Performance

**Figure 2:**
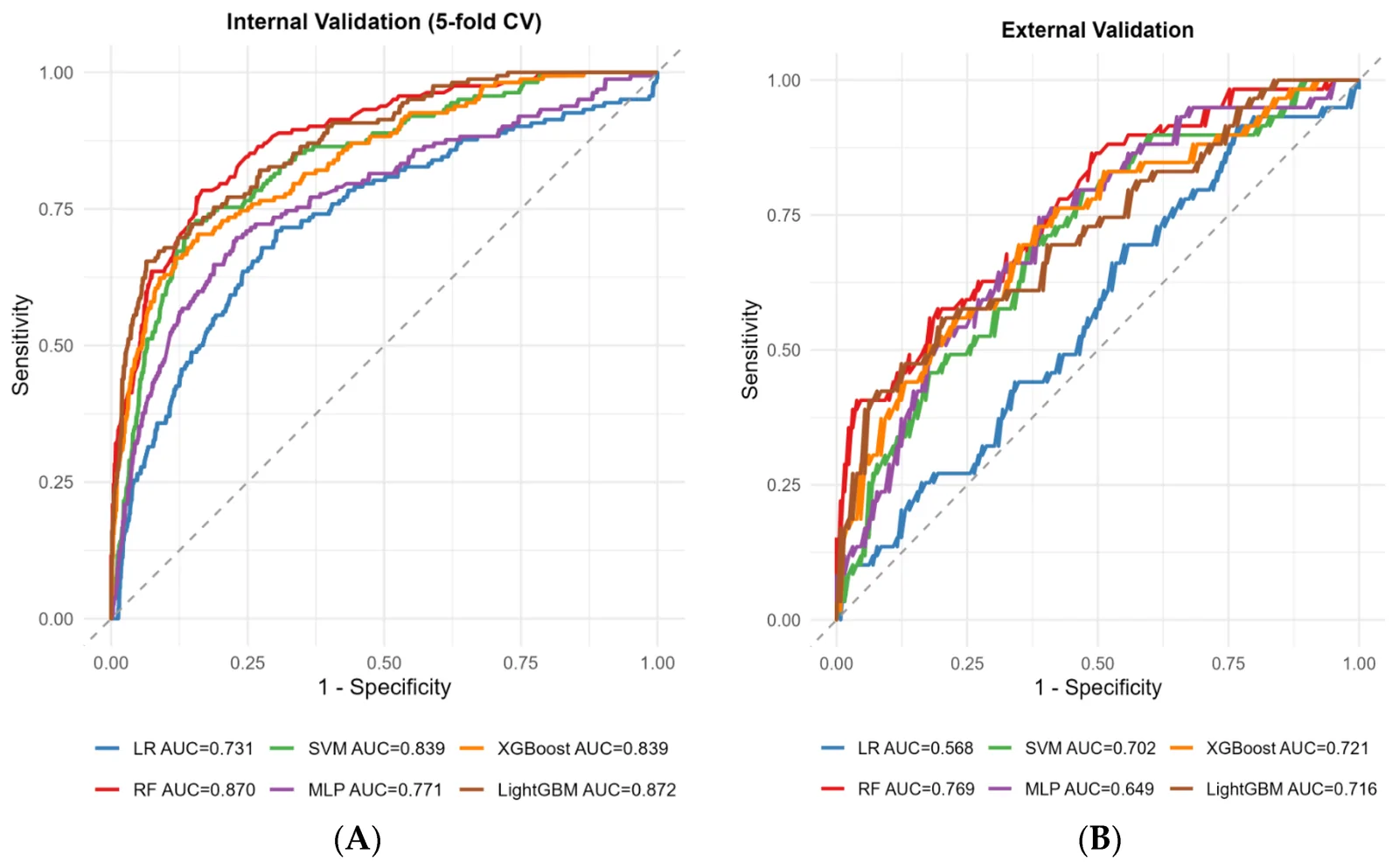
Performance of ML models in predicting RIHT. (**A**) ROC curves for the internal validation set. (**B**) ROC curves for the external validation set.

This study selected six machine learning models for RIHT prediction: Random Forest (RF), Support Vector Machine (SVM), Multi-Layer Perceptron (MLP), Logistic Regression (LR), Extreme Gradient Boosting (XGBoost), and Light Gradient Boosting Machine (LightGBM). The internal and external validation ROC curves for each model are shown in Figure 2.

Model performance was evaluated using accuracy, precision, recall, F1 score, AUC, Brier score, Brier skill score (BSS), balanced accuracy, and PR-AUC, as summarized in Table 2. LightGBM and Random Forest demonstrated the highest discrimination performance in the internal validation set, with AUCs of 0.8721 and 0.8701, respectively. SVM and XGBoost showed comparable performance, with AUCs of 0.8385 and 0.8391, respectively, whereas MLP and Logistic Regression achieved AUCs of 0.7708 and 0.7312, respectively. LightGBM achieved the highest overall performance, with an AUC of 0.8721, an F1 score of 0.5679, a balanced accuracy of 0.7031, and a PR-AUC of 0.7123.

Table 3 presents pairwise comparisons of AUCs using the DeLong test, with Bonferroni correction applied for 15 comparisons (adjusted significance threshold, *p* < 0.0033). After correction, LightGBM showed significantly better discrimination than Logistic Regression (*p* < 0.0001) and MLP (*p* < 0.0001). However, no statistically significant differences were observed between LightGBM and Random Forest (*p* = 0.6103), XGBoost (*p* = 0.0800), or SVM (*p* = 0.0471). Similarly, the difference between SVM and MLP did not reach the Bonferroni-corrected significance threshold (*p* = 0.0061). These findings suggest that although LightGBM achieved the numerically highest AUC, its performance was not statistically superior to several other machine learning models after correction for multiple comparisons.

### 3.3. Feature Importance and SHAP-Based Model Interpretation

**Figure 3:**
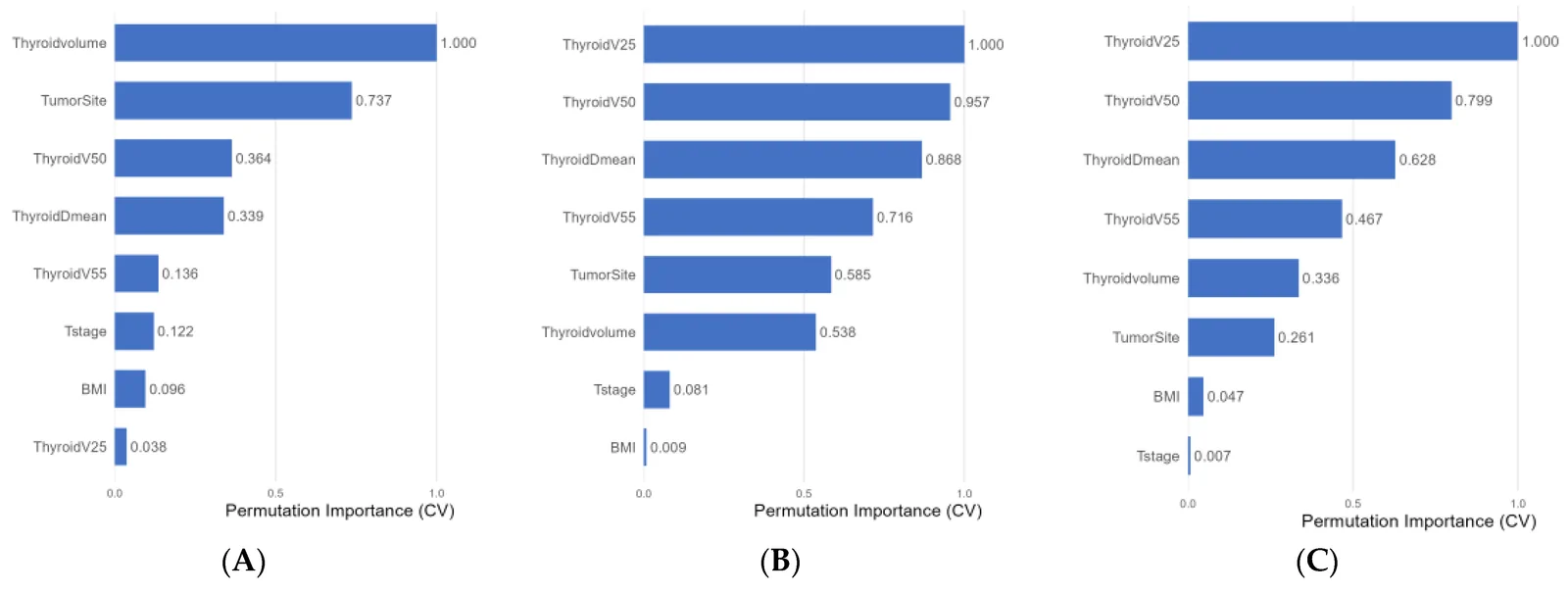

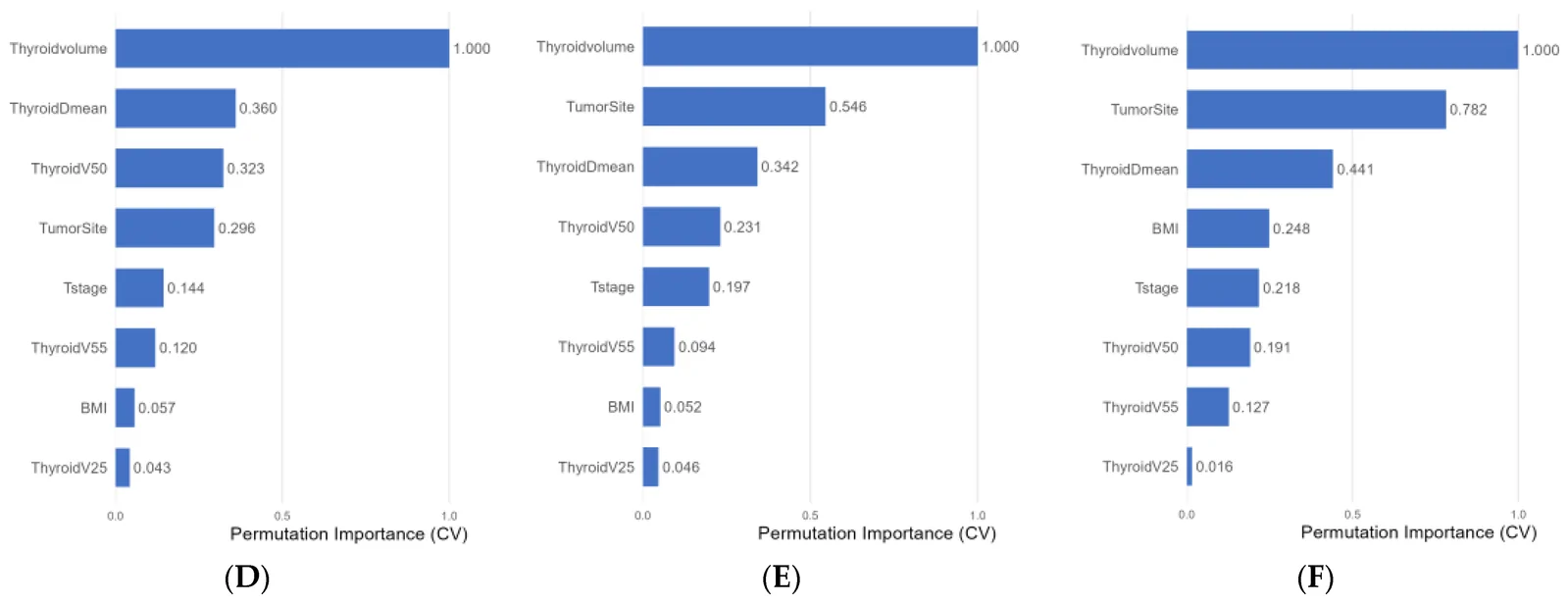
Feature importance analysis of all models. (**A**) LightGBM; (**B**) Logistic Regression; (**C**) Multi-Layer Perceptron; (**D**) Random Forest; (**E**) Support Vector Machine; (**F**) XGBoost.

**Figure 4:**
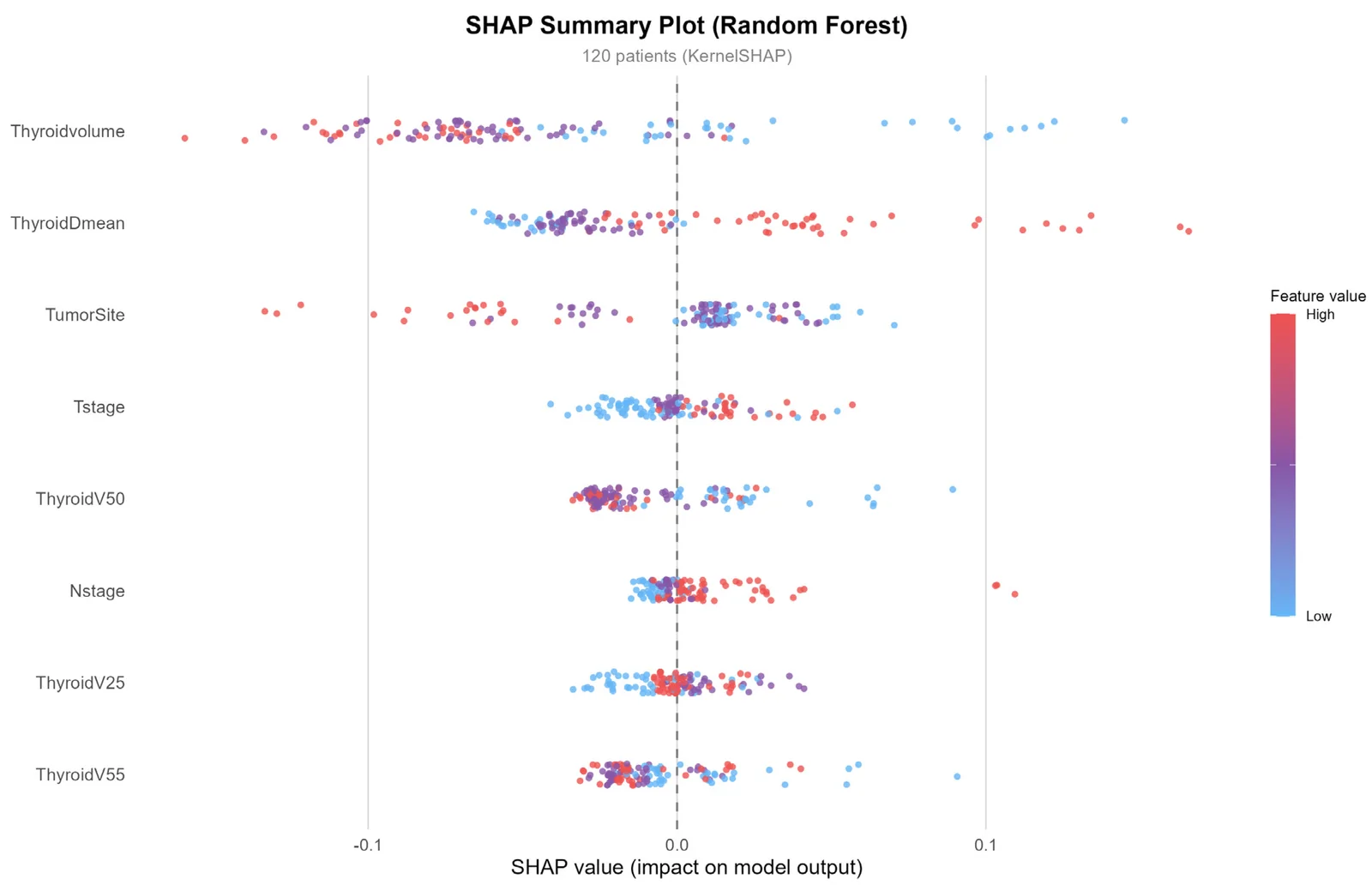
SHAP feature importance.

To further elucidate the decision-making mechanisms across different models, feature importance analyses were conducted for all machine learning algorithms. Figure 3 shows a bar chart of SHAP values, which are used to quantify and visualize the contribution of each feature to the model’s predictions. SHAP values indicate the degree of influence each feature has on the final prediction under different feature combinations. A large absolute SHAP value indicates a greater contribution of the corresponding feature to the model prediction. The top five most influential features include thyroid volume before radiotherapy, T-stage, tumor site, thyroid Dmean and V50 (Figure 4), which quantifies each variable’s contribution to the model’s output. In the SHAP plot, positive values indicate that a feature increases the predicted risk relative to the baseline, while negative values indicate a decrease; the larger the absolute value, the greater the impact on the prediction.

By analyzing the SHAP values of individual features, it is evident how the model adjusts its risk prediction based on changes in specific feature values. This detailed feature analysis not only enhances the interpretability of the model, but also provides profound insights into the role of risk factors in RIHT risk, providing valuable reference applications for clinical practice.

**Figure 5:**
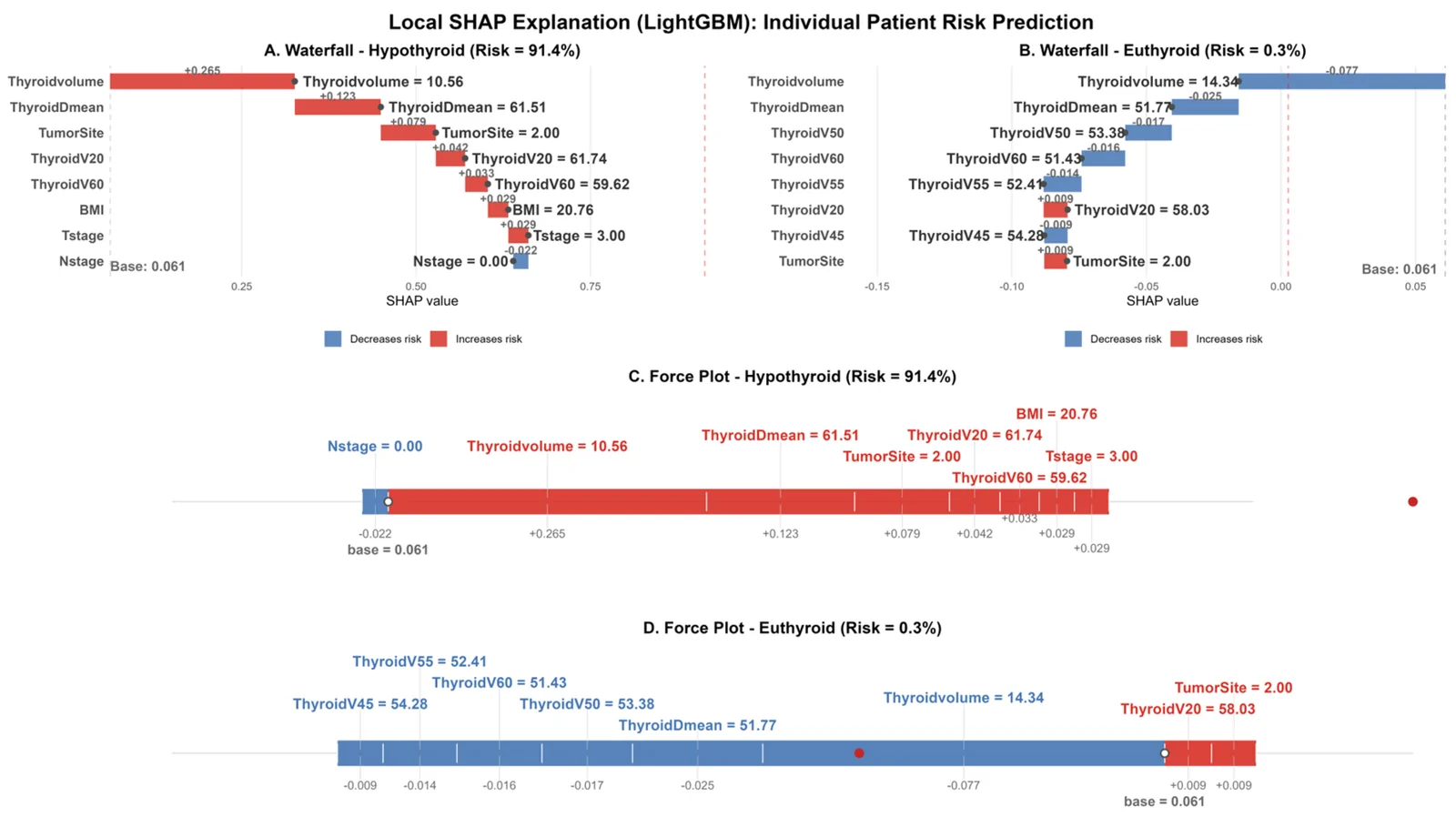
Local model explanation by the SHAP method. (**A**) Waterfall plot for one hypothyroid patient. (**B**) Waterfall plot for one euthyroid patient. (**C**) Force plot for one hypothyroid patient. (**D**) Force plot for one euthyroid patient.

Figure 5A,B are waterfall plots that show the contribution of different features to the model’s prediction output (SHAP values) for a given patient. Each bar represents a feature, with blue indicating a negative contribution to the prediction result (lowering risk) and red indicating a positive contribution (increasing risk).

Figure 5C,D are force plots that illustrate how each feature contributes to the final prediction value f(*x*), starting from the model’s baseline prediction value (base value). Blue arrows represent features that lower the prediction value, while red arrows represent features that increase the prediction value.

Figure 5A,C correspond to the same individual (hypothyroid patient). The characteristics of thyroid volume, thyroid Dmean, and tumor site are the main factors leading to increased risk, significantly increasing the predictive value of the model, indicating that patients have a higher risk of developing RIHT. The final predicted value is 91.4%, indicating that the patient has a higher risk of developing RIHT.

Figure 5B,D correspond to another individual (euthyroid patient). The thyroid volume is relatively large before radiotherapy, which reduces the risk of RIHT. The final predicted value is 0.3%, indicating that the patient has a low risk of developing RIHT.

**Figure 6:**
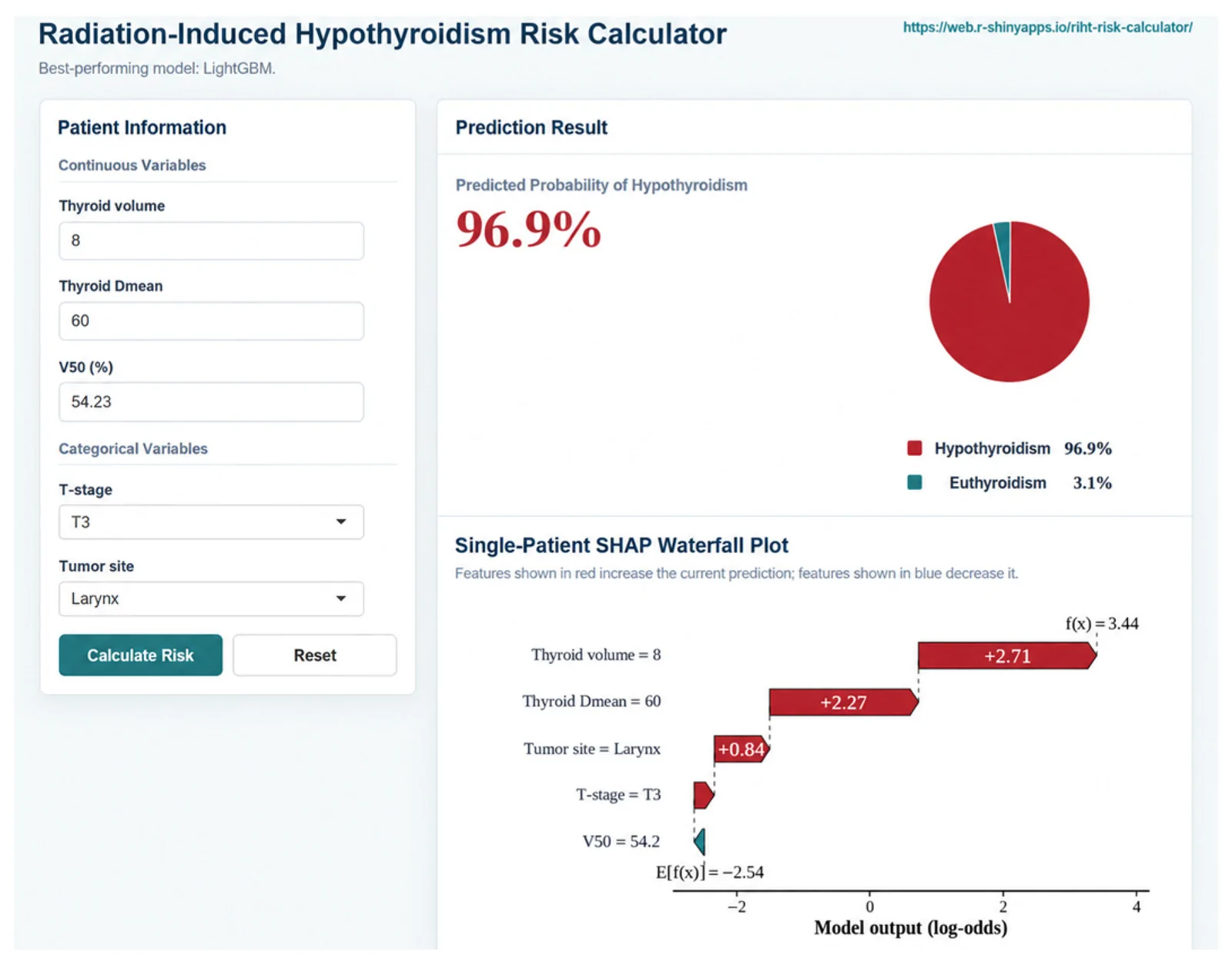
Web interface and prediction result. After filling in all necessary information, users can click the “Predict” button to perform RIHT risk prediction. The model predicts that the patient is a high-risk group for RIHT, with a probability of 96.9%. This figure shows the contribution of each feature to the final predicted score.

We developed a web application using the Shiny framework of R version 4.3.2. to dynamically predict the incidence of RIHT. This dynamic model can be accessed at https://web-r.shinyapps.io/riht-risk-calculator/ (accessed on 27 July 2026). After setting each parameter as shown in Figure 6, the prediction results are displayed as high risk or low risk for RIHT, along with the corresponding percentage. In addition, using the SHAP interpretability method, the importance of each feature is provided, with red indicating increased RIHT risk and blue indicating decreased RIHT risk. It is important to emphasize that this online calculator is intended for research and educational purposes only. It does not provide medical advice, diagnosis, or treatment recommendations. Clinical decisions should always be made by qualified healthcare professionals based on comprehensive patient evaluation.

### 3.4. External Validation

The external validation dataset was obtained from the First Affiliated Hospital of Kunming Medical University, as shown in Supplementary Table S1. This cohort included euthyroid (*n* = 202) and RIHT cases (*n* = 94). This dataset was used to further analyze the consistency of the model validation and its potential impact on RIHT prediction.

Table 4 shows that the models exhibited variable performance in the external validation cohort. SVM achieved the highest recall (0.712), whereas the remaining models showed recall values ranging from 0.458 to 0.525. In terms of discrimination, Random Forest achieved the highest AUC (0.769), followed by XGBoost (0.721), LightGBM (0.716), SVM (0.702), MLP (0.649), and Logistic Regression (0.568). These findings indicate variable discriminatory performance across models in the independent external validation cohort.

### 3.5. Calibration and Decision Curve Analysis

To further evaluate the clinical utility of the six machine learning models, we assessed calibration using calibration curves with Brier scores and performed decision curve analysis (DCA) on both the internal and external validation cohorts.

Calibration curves for the internal and external validation cohorts are shown in Supplementary Figure S2A,B. In internal validation, all models were properly calibrated, and LightGBM (Brier = 0.1046) had the lowest Brier score. In external validation, the calibration performance of all models has decreased, and all models tend to underestimate the risk of RIHT with higher prediction probabilities, indicating that recalibration may be necessary before clinical application.

Decision curve analyses for both cohorts are presented in Supplementary Figure S2C,D. In internal validation, most models provided positive net benefit across threshold probabilities. In external validation, net benefit was more limited. While the models generally outperformed the “treat none” strategy, they did not consistently exceed the “treat all” strategy across most threshold ranges, suggesting that the current models offer modest clinical utility and require further validation before implementation in clinical practice.

## 4. Discussion

RIHT is one of the common complications of radiation therapy for HNC. In its early stages, it typically lacks obvious symptoms, resulting in a large number of high-risk individuals remaining undiagnosed during treatment. HT increases the risk of atherosclerosis [18], raises the incidence and mortality rates of cardiovascular and cerebrovascular diseases, and ultimately significantly reduces patients’ quality of life [19]. According to relevant literature, the incidence of HT 2 years after radiotherapy ranges from 40% to 50%, with subclinical HT accounting for 70% and clinical HT for 30% [20]. The 2010 Report on the Quantitative Analysis of Clinical Normal Tissue Effects did not mention a dose limit for the thyroid [21]. Consequently, many current studies are dedicated to identifying risk factors for post-radiotherapy hypothyroidism in HNC patients and establishing the probability of normal tissue complications to ensure limited thyroid dose. However, no consensus has yet been reached in the relevant research. This study utilizes large-scale, multicenter retrospective data and applies various ML models to predict RIHT, with the goal of providing a more accurate predictive tool for clinical practice. This not only improves the diagnostic rate of RIHT but also offers new insights for personalized treatment and risk assessment.

ML models offer significant advantages in disease prediction [22]. In this study, six different ML models were used to predict RIHT. In our comparison of six machine learning algorithms, LightGBM showed higher numerical AUC and Brier skill scores compared to other models. However, after multiple comparisons and corrections, the difference between LightGBM and XGBoost (or Random Forest) was not statistically significant. Machine learning models may not significantly outperform other models in medium-sized clinical datasets. More importantly, several machine learning models demonstrated good discriminatory performance in internal validation, with LightGBM achieving the numerically highest AUC (0.8721), followed closely by Random Forest (0.8701). This indicates that the model not only captures the contribution of individual biomarkers to RIHT risk but also identifies interactions among different features, enabling more accurate RIHT risk prediction. LightGBM is an ensemble algorithm developed by Microsoft that provides an efficient implementation of gradient boosting algorithms [23]. The primary benefit of LightGBM is that it greatly accelerates the training process, which in many cases results in more effective models. LightGBM is built upon decision tree algorithms and employs a nested structure of boosted trees. For prediction problems, tree-boosting algorithms outperform other algorithms [24]. The LightGBM model performs exceptionally well in feature selection, model optimization, and handling complex high-dimensional data. By combining patients’ clinical baseline characteristics with dosimetric parameters, The LightGBM model achieved an AUC of 0.872 and an accuracy of 0.863 in internal validation, while maintaining moderate discrimination in the external cohort (AUC = 0.716; accuracy = 0.723).

In our study, SHAP analysis identified pre-radiotherapy thyroid volume, T-stage, thyroid Dmean, tumor site, and V50 as important contributors to model predictions. Among these variables, thyroid volume, thyroid Dmean, and tumor site also showed statistically significant associations with RIHT in the univariate analysis. Thyroid volume represents the functional reserve of the thyroid; it is influenced by gender, age, and body size, and may vary across different populations. In our study, thyroid volume and thyroid Dmean were predictive factors for RIHT. Chawalit [25] et al. found that in nasopharyngeal carcinoma patients, a small native thyroid volume (<8 cm^3^) during radiotherapy increased the probability of post-radiation thyroid injury. Liang Peng [26] found that in patients with large thyroid volumes (>20 cm^3^), radiotherapy did not appear to have a significant impact on the thyroid; however, in patients with smaller thyroid volumes (≤20 cm^3^), there was a certain probability of thyroid injury following irradiation. Additionally, many studies have shown that the risk of RIHT is dose-dependent. Many researchers believe that the mean dose to the thyroid may be the most significant dosimetric factor. A study by Zhai et al. demonstrated that Dmean influences the incidence of RIHT in nasopharyngeal carcinoma patients. When grouped by Dmean, they found that patients with Dmean > 45 Gy were nearly five times more likely to experience RIHT than those with Dmean ≤ 45 Gy [27]. While Zhai et al. reported a five-fold higher RIHT risk with Dmean > 45 Gy, we caution against using any single value as a rigid clinical cutoff. The dose–response relationship for RIHT is fundamentally continuous, and thyroid dose should be minimized following the ALARA principle rather than targeted to a specific threshold. We found that thyroid Dmean is a strong predictor of RIHT. Furthermore, in the latest international guidelines, the target threshold for thyroid Dmean is set below 50 Gy [28].

Our study found that tumor site—specifically the site of the primary tumor—is a risk factor for RIHT. Differences in radiation field coverage across various tumor sites indirectly influence the radiation dose to the thyroid, rather than simply the central dose to the thyroid. Ahmad Ameri et al. [29] found that a predictive model incorporating cancer histology, primary tumor site, V15, 30, and VS15, 30 effectively predicted post-radiation hypothyroidism, which is consistent with our results. T-stage may be a risk factor for RIHT. In the study by Ahmad Ameri et al. a large proportion of laryngeal cancer cases were early-stage (T1–T2N0) and received narrow-field irradiation covering only the primary site, largely sparing the thyroid. In contrast, most oral cancer patients have advanced primary tumors requiring therapeutic/selective neck irradiation, which inadvertently exposes the thyroid and increases the risk of RIHT. However, Sachdev et al. [30] reported that T-stage in head and neck cancer patients is not a risk factor for RIHT, and the predictive value of T-stage remains controversial; further large-scale studies are needed in the future.

With advances in radiation therapy techniques, the dose thresholds associated with RIHT in patients with HNC require further investigation. A study by Cella et al. [31] demonstrated that thyroid V30 is an independent predictor of RIHT in patients with HNC. When V30 > 62.5%, the incidence of RIHT was significantly higher in the study group compared to the control group (70.8% vs. 11.5%). Similarly, studies by Kim et al. [32] and Sachdev et al. [30] reported that thyroid V45 and V50 in HNC are independent risk factors for the development of RIHT, with thresholds of 50% and 60%, respectively. In our study, thyroid V50 was a predictor of RIHT; however, there is currently no consensus regarding the optimal dose threshold for the thyroid.

This study systematically analyzed multidimensional data from RIHT patients and validated the potential of machine learning models, such as XGBoost and LightGBM, for predicting RIHT. Notably, the LightGBM model demonstrated good predictive performance in internal validation and maintained moderate discriminatory performance in the external validation cohort. Combined with SHAP-based interpretability analysis, the clinical application of this model becomes more transparent and reliable, providing strong support for RIHT detection. We acknowledge a limitation of our SHAP analysis: the high correlation among Vx dose–volume parameters (Supplementary Figure S1) can lead to unstable feature importance estimates, as SHAP values may be arbitrarily distributed among correlated features. Therefore, we caution against overinterpreting the relative importance of V50 vs. V55 or any other individual Vx threshold. Instead, the key message is that mid-to-high dose–volume parameters collectively (V40–V60) are important predictors of RIHT, reflecting the cumulative effect of thyroid irradiation rather than a specific dose threshold.

However, our study has several limitations. First, the follow-up period was short, lasting only 12 months. Future studies should extend the follow-up duration. Second, the sample size was small; future research should aim to reduce the risk of overfitting by expanding the sample size—particularly through further multicenter data collection—and by adopting more rigorous model evaluation methods, such as incorporating hematological indicators. Third, to address missing data in RIHT, future studies will collect more comprehensive case data over a longer follow-up period to ensure the integrity and reliability of model training. Fourth, our study only included patients who completed 12 months of follow-up without disease progression or death. While this design ensured a clean assessment of radiation-attributable hypothyroidism, it introduces a selection bias toward patients with better prognosis. Patients with aggressive disease who progress or die early are not represented in our cohort. Therefore, our model’s generalizability may be limited to HNC patients with a life expectancy of at least 12 months. Future prospective studies with larger sample sizes, regular event monitoring, and complete cause-of-death data should employ competing risk models (e.g., cumulative incidence function with death as a competing event) to validate and extend our findings. Fifth, our study used a binary outcome at the fixed 12-month time point based on protocol-specified TSH measurements. We did not have sufficient data on the exact timing of RIHT onset (TSH was not measured continuously), nor did we have a large enough sample to produce well-calibrated individual risk probabilities. Sixth, the limited number of RIHT events (*n* = 54) in the development cohort constrains model complexity. Although repeated 5-fold cross-validation was used to improve the robustness of internal performance estimation, our results should be interpreted as exploratory. External validation in larger cohorts is essential before clinical translation. Future prospective studies with regular TSH monitoring (e.g., every 1–2 months) and larger cohorts are needed to perform time-to-event analyses and provide calibrated risk estimates for clinical use. At the same time, with advances in health monitoring technology, real-time dynamic monitoring combined with ML models will open new avenues for early warning and personalized treatment of RIHT.

## 5. Conclusions

This study developed and validated several machine learning models for predicting RIHT in patients with HNC following radiotherapy. Among the six algorithms compared, tree-based ensemble methods (LightGBM, XGBoost, and Random Forest) demonstrated robust predictive performance in internal validation, collectively outperforming the traditional logistic regression model. While LightGBM showed numerically higher AUC, its advantage over other tree-based methods was not statistically significant after correction for multiple comparisons. Through SHAP interpretability analysis, pre-treatment thyroid volume, T-stage, mean thyroid dose, tumor site, and V50 were identified as key contributors to RIHT prediction, thereby enhancing the model’s clinical transparency and interpretability.

While the models showed promising internal performance, the external validation recall suggests that approximately half of RIHT cases would be missed at the current threshold. The current models are therefore not yet suitable for clinical decision support and should be viewed as exploratory tools requiring further refinement and validation in larger cohorts before clinical translation. This method is expected to reduce the incidence of RIHT and improve the long-term quality of life for head and neck cancer patients. Future studies should expand the sample size, extend the follow-up period, and incorporate additional hematological and imaging parameters to continuously optimize model performance and facilitate its clinical implementation.

## Figures and Tables

**Table 1:** Baseline demographic, clinical, and dosimetric characteristics of the Harbin cohort stratified by thyroid function status.

Variables	Euthyroid (*N* = 202)	Hypothyroid (*N* = 54)	*p*
Age	58.75 ± 11.43	58.04 ± 9.34	0.720
BMI	21.62 ± 3.40	21.63 ± 2.17	0.639
Thyroid Volume	13.21 ± 2.69	11.72 ± 2.70	<0.001
V20	75.26 ± 24.57	77.51 ± 20.49	0.386
V25	72.99 ± 24.87	75.82 ± 21.23	0.354
V30	70.66 ± 24.89	73.90 ± 22.07	0.290
V35	67.72 ± 25.04	71.19 ± 22.26	0.280
V40	63.41 ± 25.21	66.30 ± 24.58	0.331
V45	57.79 ± 26.49	60.75 ± 25.56	0.195
V50	51.04 ± 26.65	52.63 ± 27.40	0.456
V55	40.74 ± 27.83	45.57 ± 30.06	0.215
V60	33.65 ± 26.96	38.26 ± 27.37	0.126
Dmean	48.43 ± 12.93	52.40 ± 12.73	0.009
Gender			0.591
Female	50 (24.8%)	11 (20.4%)	
Male	152 (75.2%)	43 (79.6%)	
Clinical Stage			0.048
I	36 (17.8%)	7 (13.0%)	
II	32 (15.8%)	4 (7.4%)	
III	80 (39.6%)	18 (33.3%)	
IV	54 (26.7%)	25 (46.3%)	
T stage			0.077
T1	30 (14.9%)	9 (16.7%)	
T2	68 (33.7%)	9 (16.7%)	
T3	62 (30.7%)	19 (35.2%)	
T4	42 (20.8%)	17 (31.5%)	
N stage			0.052
N0	68 (33.7%)	12 (22.2%)	
N1	48 (23.8%)	11 (20.4%)	
N2	78 (38.6%)	24 (44.4%)	
N3	8 (4.0%)	7 (13.0%)	
Chemotherapy			0.444
No	100 (49.5%)	23 (42.6%)	
Yes	102 (50.5%)	31 (57.4%)	
Tumor site			<0.001
Nasopharynx	56 (27.7%)	18 (33.3%)	
Larynx	79 (39.1%)	31 (57.4%)	
Oropharynx	26 (12.9%)	2 (3.7%)	
Oral cavity	3 (1.5%)	3 (5.6%)	
Nasal cavity and sinus	29 (14.4%)	0 (0.0%)	
Salivary gland	9 (4.5%)	0 (0.0%)	

**Table 2:** Evaluation metrics for the internal validation set.

Metric	Logistic Regression	Random Forest	SVM	MLP	XGBoost	LightGBM
Accuracy	0.7969	0.8477	0.832	0.8177	0.8398	0.8633
Precision	0.5288	0.9091	0.6737	0.5917	0.791	0.8519
Recall	0.3395	0.3086	0.3951	0.4383	0.3272	0.4259
F1	0.4135	0.4608	0.4981	0.5035	0.4629	0.5679
AUC	0.7312	0.8701	0.8385	0.7708	0.8391	0.8721
Brier	0.1495	0.1143	0.119	0.1485	0.1144	0.1046
BSS	0.1017	0.3136	0.2848	0.1077	0.3126	0.3713
Balanced_Accuracy	0.6293	0.6502	0.672	0.6787	0.652	0.7031
PR_AUC	0.4268	0.7105	0.5774	0.5033	0.6549	0.7123

**Table 3:** The AUC DeLong test results. Bonferroni correction was applied for 15 pairwise comparisons; statistical significance was defined as *p* < 0.0033.

Model 1	Model 2	AUC1_CI	AUC2_CI	*p*-Value
LR	RF	0.731 (0.684–0.778)	0.870 (0.849–0.909)	<0.0001
LR	SVM	0.731 (0.684–0.778)	0.839 (0.803–0.874)	<0.0001
LR	MLP	0.731 (0.684–0.778)	0.771 (0.726–0.815)	0.1475
LR	XGBoost	0.731 (0.684–0.778)	0.839 (0.803–0.875)	<0.0001
LR	LightGBM	0.731 (0.684–0.778)	0.872 (0.841–0.903)	<0.0001
RF	SVM	0.870 (0.849–0.909)	0.839 (0.803–0.874)	0.0267
RF	MLP	0.870 (0.849–0.909)	0.771 (0.726–0.815)	<0.0001
RF	XGBoost	0.870 (0.849–0.909)	0.839 (0.803–0.875)	0.005
RF	LightGBM	0.870 (0.849–0.909)	0.872 (0.841–0.903)	0.6103
SVM	MLP	0.839 (0.803–0.874)	0.771 (0.726–0.815)	0.0061
SVM	XGBoost	0.839 (0.803–0.874)	0.839 (0.803–0.875)	0.9765
SVM	LightGBM	0.839 (0.803–0.874)	0.872 (0.841–0.903)	0.0471
MLP	XGBoost	0.771 (0.726–0.815)	0.839 (0.803–0.875)	0.0081
MLP	LightGBM	0.771 (0.726–0.815)	0.872 (0.841–0.903)	<0.0001
XGBoost	LightGBM	0.839 (0.803–0.875)	0.872 (0.841–0.903)	0.08

**Table 4:** Model evaluation metrics for external validation.

Metric	Logistic Regression	Random Forest	SVM	MLP	XGBoost	LightGBM
Accuracy	0.5532	0.7234	0.6223	0.6649	0.7181	0.7234
Precision	0.3418	0.5686	0.4375	0.4697	0.56	0.5686
Recall	0.4576	0.4915	0.7119	0.5254	0.4746	0.4915
F1-Score	0.3913	0.5273	0.5419	0.496	0.5138	0.5273
AUC	0.5675	0.7688	0.702	0.6489	0.7212	0.7157
Brier	0.2677	0.1734	0.2757	0.2659	0.1941	0.2078
BSS	−0.2432	0.1949	−0.2805	−0.2346	0.0985	0.035
Balanced_Accuracy	0.5273	0.6605	0.6466	0.6271	0.652	0.6605
PR_AUC	0.3734	0.6443	0.4908	0.4878	0.5515	0.5746

## Data Availability

The data presented in this study are available upon request from the corresponding author due to privacy and ethical restrictions regarding clinical patient data.

## Supplementary Materials

The following supporting information can be downloaded at https://www.mdpi.com/article/10.3390/cancers18172838/s1, Figure S1: Spearman correlation matrix of dose–volume parameters (V20–V60 and thyroid Dmean). Correlation coefficients ≥ 0.90 are observed among V30–V60, indicating high multicollinearity. Thyroid Dmean is also highly correlated with V40–V60 (r = 0.85–0.92). These correlations suggest that individual Vx thresholds should be interpreted as a cluster rather than independent predictors; Figure S2: Calibration curves and decision curve analysis for six machine learning models. (A) Calibration curves for the six models in the internal validation cohort. (B) External validation cohort. Calibration curves in the external validation cohort. (C) Decision curves in the internal validation cohort. (D) Decision curves in the external validation cohort; Table S1: External Validation Data Baseline Table; Table S2. Default hyperparameter configurations for the six machine learning models.

## Abbreviations

The following abbreviations are used in this manuscript:

RIHT	Radiation-Induced Hypothyroidism
IMRT	Intensity-Modulated Radiotherapy
HNC	Head and Neck Cancer
Dmean	the mean dose of thyroid
NPC	Nasopharyngeal carcinoma
DVHs	Dose–volume histograms
TSH	Thyroid-stimulating hormone
FT3	Free triiodothyronine
FT4	Free thyroxine
ROC	Receiver operating characteristic
