# Supplementary material for: Interpretable Machine Learning for Predicting Radiation-Induced Hypothyroidism in Head and Neck Cancer: A Dual-Center Retrospective Study
Source: Cancers (Basel). 2026 Sep 2;18(17):2838. doi: 10.3390/cancers18172838 (PMC13564486; doi:10.3390/cancers18172838)
Supplement: Supplementary file 1 [file cancers-18-02838-s001.zip › cancers-4495219-supplementary.pdf]

Supplementary Material

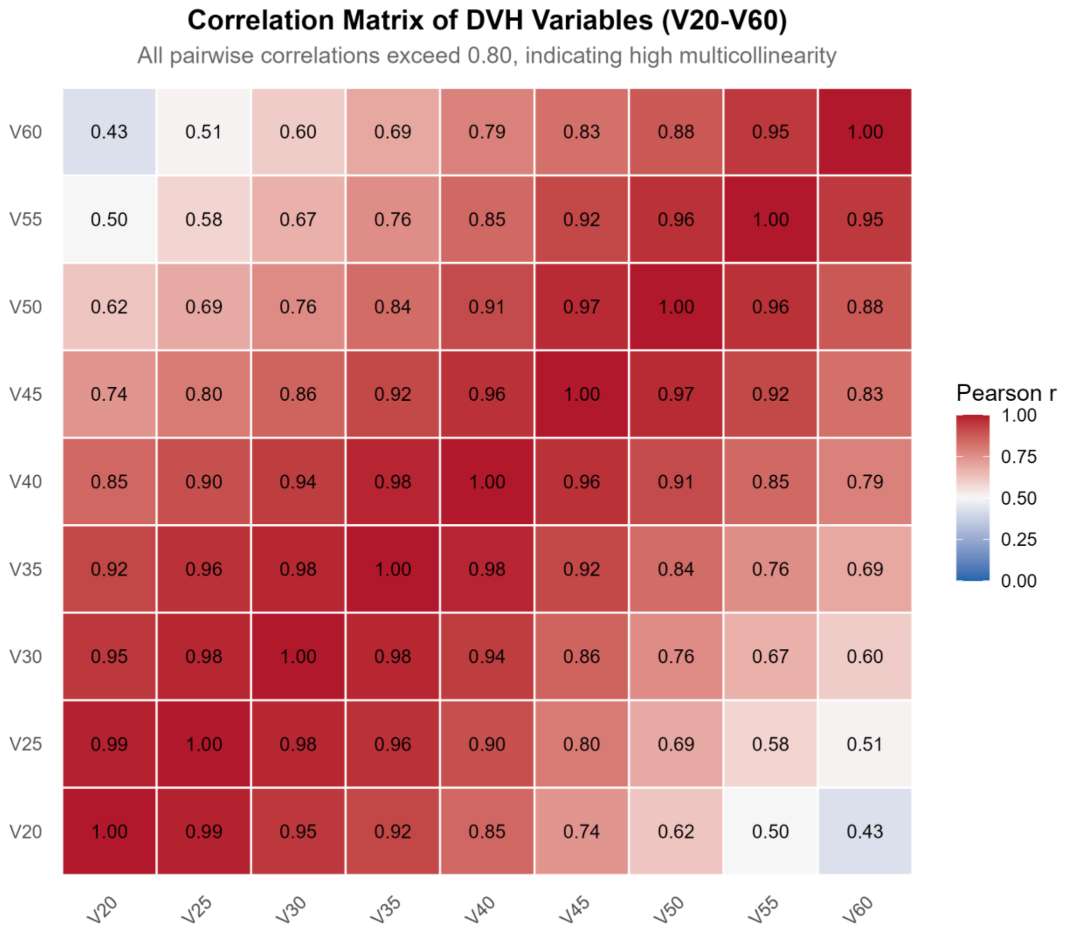

Figure S1. Spearman correlation matrix of dose–volume parameters (V20–V60 and thyroid Dmean). Correlation coefficients ≥0.90 are observed among V30–V60, indicating high multicollinearity. Thyroid Dmean is also highly correlated with V40–V60 (r = 0.85–0.92). These correlations suggest that individual Vx thresholds should be interpreted as a cluster rather than independent predictors.

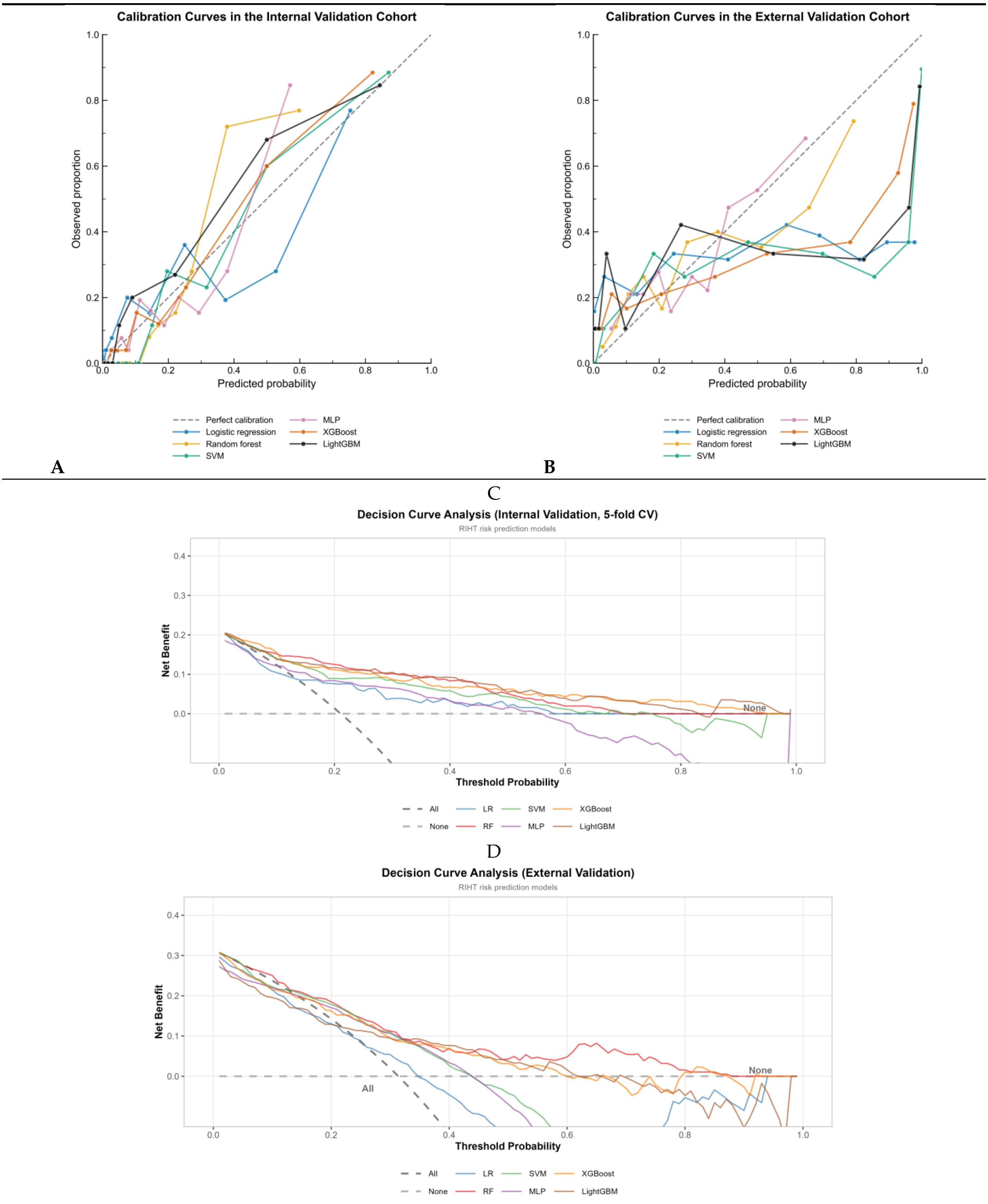

Figure S2. Calibration curves and decision curve analysis for six machine learning models. (A) Calibration curves for the six models in the internal validation cohort. (B) External validation cohort. Calibration curves in the external validation cohort. (C) Decision curves in the internal validation cohort. (D) Decision curves in the external validation cohort.

Table S1. External Validation Data Baseline Table.

Variables	Euthyroid（N=202）	Hypothyroid(N=94)	*P*
Age	52.59±11.55	53.3±10.46	0.600
BMI	21.47	21.29	0.658
Thyroid Volume	11.74	10.07	<0.001
V20	60.29	62.31	0.033
V25	60.14	61.34	0.209
V30	59.13	59.93	0.415
V35	58.10	59.90	0.081
V40	56.80	58.37	0.123
V45	56.21	57.48	0.250
V50	52.75	58.99	<0.001
V55	54.64	56.29	0.139
V60	53.93	54.94	0.364
Thyroid Dmean	53.40	54.95	0.281
Gender			0.009
Female	35	5	
Male	167	89	
Clinical Stage			0.135
I	18	4	
II	30	13	
III	45	14	
IV	109	63	
T stage			0.224
T1	33	14	
T2	43	13	
T3	49	20	
T4	77	47	
N stage			0.037
N0	55	13	
N1	45	32	
N2	57	27	
N2	45	22	
Chemotherapy			0.928
No	39	17	
Yes	163	77	
Tumor type			0.551
Nasopharynx	100	53	
Larynx	42	19	
Oropharynx	21	8	
Oral cavity	9	6	
Nasal cavity and sinus	13	2	
Salivary gland	17	6	

All models were trained using the default hyperparameters from their respective Python libraries. The stability of these default parameters was verified using 5-fold cross-validation on the training set, which yielded low variance in AUC (standard deviation < 0.03 for all models). A limited grid search on LightGBM (exploring n_estimators in [50, 100, 200] and max_depth in [3, 5, 7]) resulted in only a marginal AUC improvement (+0.01), confirming that default parameters are adequate for this dataset and avoiding the risk of overfitting to the validation set. For SVM and MLP, feature scaling was applied using StandardScaler prior to model training.

Table S2. Default hyperparameter configurations for the six machine learning models.

Model	Library & Version	Hyperparameter	Default Value
**LightGBM**	lightgbm v3.3	n_estimators	100
		max_depth	-1 (no limit)
		learning_rate	0.1
		num_leaves	31
		subsample	1.0
		colsample_bytree	1.0
		min_child_samples	20
		reg_alpha	0.0
		reg_lambda	0.0
**XGBoost**	xgboost v1.7	n_estimators	100
		max_depth	6
		learning_rate	0.3
		subsample	1.0
		colsample_bytree	1.0
		min_child_weight	1
		gamma	0
		reg_alpha	0
		reg_lambda	1
**Random Forest**	scikit-learn v1.2	n_estimators	100
		max_depth	None

Model	Library & Version	Hyperparameter	Default Value
		min_samples_split	2
		min_samples_leaf	1
		max_features	'sqrt'
		bootstrap	True
		criterion	'gini'
**Support Vector Machine (SVM)**	scikit-learn v1.2	kernel	'rbf'
		C	1.0
		gamma	'scale'
		tol	1e-3
		class_weight	None
**Logistic Regression (LR)**	scikit-learn v1.2	penalty	'l2'
		C	1.0
		solver	'lbfgs'
		max_iter	100
		class_weight	None
**Multi-Layer Perceptron (MLP)**	scikit-learn v1.2	hidden_layer_sizes	(100,)
		activation	'relu'
		solver	'adam'
		alpha	0.0001
		learning_rate	'constant'
		max_iter	200

Model	Library & Version	Hyperparameter	Default Value
		early_stopping	False
